# Endogenous cathelicidin production limits inflammation and protective immunity to *Mycobacterium avium* in mice

**DOI:** 10.1002/iid3.7

**Published:** 2013-10-31

**Authors:** José Carlos Santos, Sandro Silva-Gomes, João Pedro Silva, Miguel Gama, Gustavo Rosa, Richard L Gallo, Rui Appelberg

**Affiliations:** 1 Instituto de Biologia Molecular e Celular (IBMC), University of Porto4150-180, Porto, Portugal; 2 Centre of Biological Engineering, Universidade do MinhoBraga, Portugal; 3 Department of Medicine, Division of Dermatology, University of California9500 Gilman Drive, San Diego, CA, 92093-0612, USA

**Keywords:** Antimicrobial peptides, cathelicidin, macrophage

## Abstract

The production of antimicrobial peptides, such as the cathelicidins, plays a prominent role in the innate immune response against microbial pathogens. Cathelicidins are widely distributed amongst living organisms, and the antimicrobial peptides generated by proteolysis of the precursor forms are typically cationic and α-helical, a structure that facilitates their interaction and insertion into anionic bacterial cell walls and membranes, causing damage and promoting microbial death. Here, we found that mouse cathelicidin (*Camp*) expression was induced in bone marrow-derived macrophages by infection with *Mycobacterium avium* in a TLR2- and TNF-dependent manner. However, the endogenous production of the cathelin-related antimicrobial peptide (CRAMP) was not required for the bacteriostasis of *M. avium* either in primary cultures of macrophages or *in vivo*, as shown by the use of CRAMP-null mice. In contrast, the lack of *Camp* led to a transient improvement of *M. avium* growth control in the spleens of infected mice while at the same time causing an exacerbation of the inflammatory response to infection. Our data highlight the anti-inflammatory effects of CRAMP and suggests that virulent mycobacteria may possess strategies to escape its antimicrobial activity.

## Introduction

Innate immunity provides the first line of defense against infection by pathogenic microorganisms. An important branch of innate immunity is composed by antimicrobial peptides (AMPs), a group of unique and diverse molecules with a wide range of microbicidal activity [1,2]. Among these AMPs, cathelicidins are found in every mammal that has been examined, and can be expressed in circulating neutrophils [3], epithelial cells of the skin [4], urinary and gastrointestinal tracts [5,6], blood-brain barrier, meninges [7] as well as in bone marrow-derived macrophages (BMMφ) [8]. Only one cathelicidin encoding gene, *CAMP*, has been found in humans [9]. This is highly homologous to the single mouse cathelicidin gene, *Camp* (also known as *CnLp*) [10]. Cathelicidins are synthesized as precursors in a pre-pro-peptide form that is first cleaved into a pro-peptide, designated pro-LL-37 or hCAP18 in humans and pro-CRAMP (cathelin-related antimicrobial peptide) in mice, and then to the mature bioactive peptide LL-37 or CRAMP, respectively [11,12]. Both LL-37 and CRAMP are amphipathic, α-helical peptides, with a net positive charge of (+6) [10,13] that preferentially binds to negatively charged groups of the outer leaflet of the bacterial envelope. This leads to displacement of lipids, alteration of membrane structure and to the creation of a physical pore in the membrane causing bacterial contents to leak out [14].

Cathelicidin expression is differentially regulated by distinct pathogens upon infection. Whereas some microorganisms such as *Shigella flexneri* and *Vibrio cholerae* inhibit cathelicidin production [15,16], others like Group A *Streptococcus* [4], *Salmonella typhimurium* [8], and *Neisseria meningitidis* [7], upregulate its expression. This points to an important role of cathelicidin in immunity, which was confirmed by the use of *Camp*-deficient mouse models. CRAMP-KO mice have impaired immunity to Group A *Streptococcus*, with larger and persistent skin infections [17], and are more susceptible to uropathogenic *Escherichia coli* and meningococcal infection than wild-type mice [5,7]. In addition to its direct antimicrobial activity, cathelicidins were suggested to act as multifunctional mediators that modify the local inflammatory response and activate adaptive immunity [18–20].

Pathogenic mycobacteria cause several long-term infections in their hosts with variable outcomes depending on the virulence of the bacteria as well on the host's immune condition [21,22]. Mice have been used to model such infections and dissect protective mechanisms, namely the effector molecules of mycobacterial control or killing. There is little information about the immunological role of AMPs, namely cathelicidin, during mycobacterial infections. We recently showed that in vitro and *in vivo* killing of a mycobacterial saprophyte involves cathelicidin-dependent mechanisms [23]. In axenic conditions, killing of virulent *Mycobacterium tuberculosis* can also be induced by treatment with AMPs. Finally, cathelicidin-dependent killing was shown to be important in *M. tuberculosis* killing in human monocytes, by a mechanism dependent on TLR2/1 and on the vitamin D pathway [24,25]. Here we used *Mycobacterium avium*, a mycobacterial species that is pathogenic for mice, to address the role of cathelicidin in the control of infection. Like other pathogenic mycobacteria, *M. avium* resides primarily within macrophages, which function both as the primary defensive host cell and the main site of bacterial replication [21]. *M. avium*, unlike other mycobacteria, is highly resistant to the classical killing mechanisms that involve oxidative damage and may be useful in identifying additional killing mechanisms in macrophages.

We show here that *M. avium* induces rapid upregulation of the CRAMP-encoding gene, in a TLR2- and TNF-dependent manner, and that CRAMP production is not required for the control of *M. avium* growth in in vitro cultured macrophages. *In vivo*, CRAMP-deficient mice exhibited improved clearance of the infection and presented an exacerbated immune inflammatory response, as compared to wild-type animals.

## Results

### *M. avium* infection leads to upregulation of cathelicidin expression

**Figure 1:**
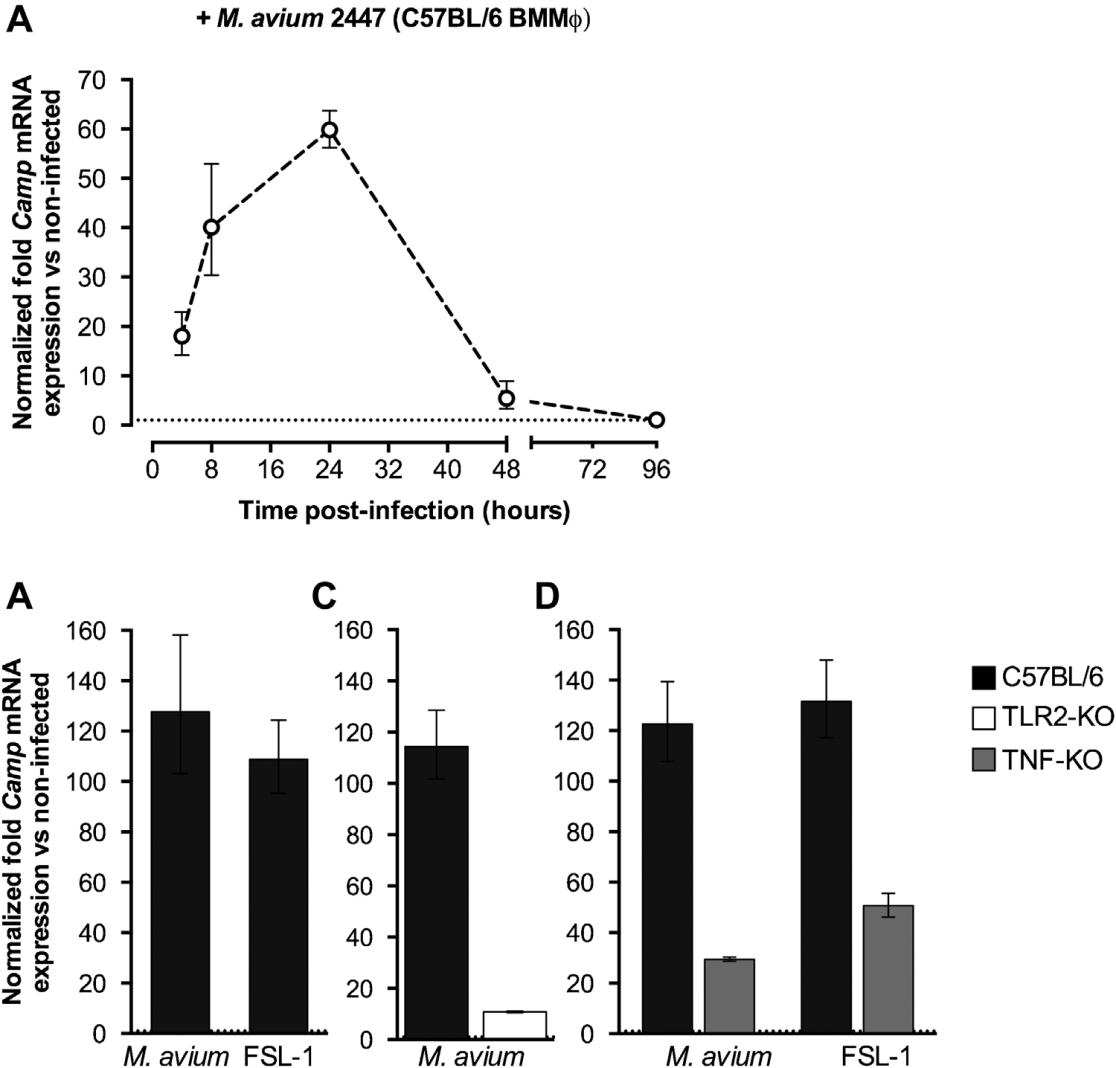
Expression of the CRAMP-coding gene in BMMφ is altered by TLR2 and TNF stimulation, after *M. avium* infection. Levels of *Camp* mRNA were determined by qPCR in cells derived from C57BL/6 mice, at different time points after infection with *M. avium* 2447 (A) or 24 h upon bacterial infection or 0.001 µg/mL FSL-1 stimulation (B). *Camp* expression after *M. avium* infection is impaired in cells derived from TLR2- (C) or TNF-deficient mice (D). Induction of *Camp* expression upon TLR2 stimulation with 0.001 µg/mL FSL-1 is ablated in BMMφ derived from TNF-deficient mice (D). Values represent the mean fold expression relative to non-infected or untreated control cells, obtained from triplicate cultures from one representative experiment.

Pathogens modulate cathelicidin expression in different ways, either by negative or positive regulation. To determine in which way infection of murine macrophages with *M. avium* regulates the expression of the CRAMP-encoding gene, bone marrow macrophages (BMMφ) isolated from C57BL/6 mice were infected and the levels of *Camp* mRNA were assessed by quantitative RT-PCR (qPCR). Infection was shown to quickly induce upregulation of cathelicidin expression, starting at 4 h post-infection, with a maximum level of 60-fold increase 24 h after *M. avium* contact (Fig. 1A).

### *M. avium*-induced cathelicidin expression is dependent on TLR2 and TNF

In order to determine cellular mechanisms involved in the induction of *Camp* upon infection with *M. avium* in BMMφ, we started by analysing if TLR2 is involved, given that this bacterium acts as a TLR2 agonist [21]. Activation of BMMφ with the TLR2/6 agonist FSL-1 resulted in upregulation of the CRAMP-encoding gene in a similar way to infection with *M. avium* (Fig. 1B), suggesting that mycobacterial infection might induce *Camp* expression in a TLR2-dependent manner. To test this, BMMφ from TLR2-deficient mice were then infected with *M. avium* and *Camp* mRNA levels were compared by qPCR to those observed in cells from wild-type C57BL/6 animals. As shown in Figure 1C, cathelicidin expression was strongly impaired in TLR2-null macrophages, demonstrating that the induction of *Camp* mRNA upon infection with *M. avium* depends on the activation of TLR2.

One important player in the control of mycobacterial infections by macrophages is TNF production [26,27], and this cytokine strongly induces cathelicidin expression in BMMφ (Fig. S1). Given that TNF production by activated macrophages is partially TLR2-dependent [28], we next tested the ability of TNF-deficient BMMφ to express cathelicidin upon infection with *M. avium* or TLR2 activation. Mouse macrophages that do not produce TNF have impaired *Camp* mRNA production upon infection or activation of TLR2, when compared to wild-type cells (Fig. 1D). This suggests that macrophage TNF production upon stimulation of TLR2 by *M. avium* is important to induce upregulation of cathelicidin. Together, these results indicate that *M. avium* induces expression of the CRAMP-coding gene, in BMMφ, via activation of the TLR2 pathway and production of TNF.

### Upregulation of cathelicidin is not required for the control of *M. avium* growth in mouse macrophages

**Figure 2:**
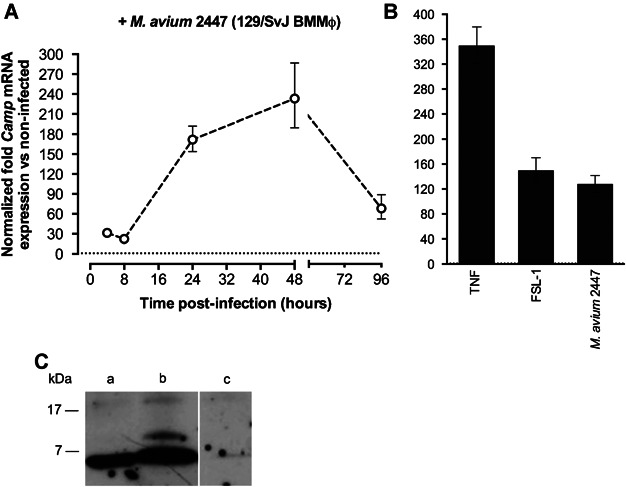
Expression of the CRAMP-coding gene in BMMφ derived from 129/SvJ mice. Cells were infected with *M. avium* 2447 and the levels of *Camp* mRNA were determined at different time points (A). *Camp* expression was quantified 48 h after 50 U/mL TNF or 0.001 µg/mL FSL-1 stimulation or bacterial infection (B). Values were determined by qPCR and represent the mean fold expression relative to non-infected or untreated control cells. Detection of the CRAMP peptide in BMMφ (C). Protein extracts (30 µg) from *Camp*-expressing (lanes a and b) or *Camp*-deficient (lane c) macrophages were analysed by Western blot using a CRAMP(1-39) antibody. Cells were either left non-infected (a) or infected with *M. avium* 2,447 (b, c). Data were obtained from triplicate cultures from one representative experiment out of a total of three experiments.

We next investigated the role of endogenous CRAMP in the control of infection by *M. avium*, using wild-type and CRAMP-deficient BMMφ. Given that available CRAMP-KO mice were on a 129/SvJ background, we first determined by qPCR the levels of cathelicidin expression in BMMφ upon infection with *M. avium*. In cells from this mouse strain infection was also shown to induce upregulation of the CRAMP-coding gene, but differed from C57BL/6 cells by having higher levels at 48 h post-infection (Fig. 2A). Treatment with FSL-1 led to a similar induction of *Camp* expression, also suggesting that its induction by *M. avium* is TLR2-dependent (Fig. 2B). Moreover, activation of murine macrophages with TNF induced high cathelicidin expression, as shown in Figure 2B. We were also able to show here that *M. avium*-infected BMMφ produce cathelicidin at the protein level, as seen by the presence of both pro- and mature-CRAMP (18 and 5 kDa, respectively) by Western blot analysis (Fig. 2C).

**Figure 3:**
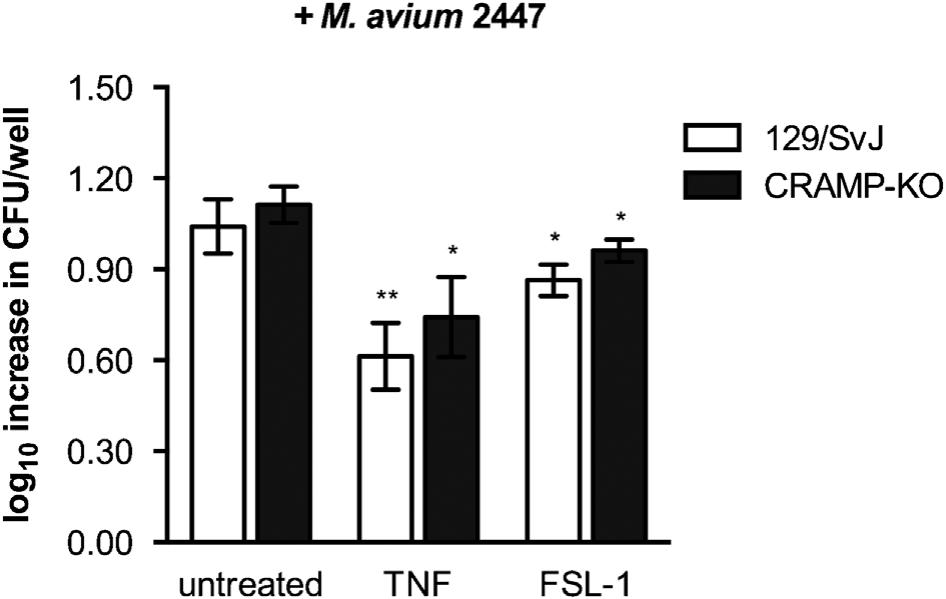
Comparison between the intracellular growth of *M. avium* 2,447 inside BMMφ from 129/SvJ or CRAMP-KO mice, for 7 days. Macrophages were infected with 10^6^ CFU per well and were either left untreated or were treated with TNF (50 U/mL) or FSL-1 (0.001 µg/mL). The difference of log_10_ CFU per well between days 0 and 7 post-infection was designated log_10_ increase CFU. The results shown are the mean log_10_ increase CFU per well ±1 SD from three wells for each condition. One representative experiment is depicted from a total of three experiments. Statistics is relative to untreated BMMφ from the same genetic background.

Treatment of BMMφ with TNF or FSL-1 was previously shown to restrict *M. avium* growth [27,28] and we demonstrate here that these stimuli induce the upregulation of the CRAMP-encoding gene. Therefore, to address the significance of cathelicidin synthesis and upregulation by BMMφ, we measured the intramacrophagic growth of *M. avium* within wild-type and CRAMP-deficient macrophages to evaluate whether CRAMP mediated the TNF- and FSL-1-induced mycobacteriostasis. BMMφ from 129/SvJ and CRAMP-deficient mice were infected with *M. avium* at a multiplicity of infection of two bacteria per macrophage, for 4 h. Infected cells were treated daily with TNF until day 4 post-infection, or with a single dose of FSL-1 just after infection, and the mycobacterial growth was then measured at different time-points for up to 7 days, by counting CFUs. Both TNF treatment and TLR2 activation with FSL-1 decreased the observed increase in *M. avium* burden (Fig. 3). Wild-type and CRAMP-deficient BMMφ responded in a similar way and, as depicted in Figure 3, the mycobacterial load at day 7 was not affected by the lack of CRAMP production.

These results show that the absence of the antimicrobial peptide CRAMP does not affect the permissiveness of BMMφ to *M. avium* growth, and that macrophages can inhibit intracellular mycobacterial growth upon cytokine treatment even when they are unable to produce cathelicidin.

### Cathelicidin-derived peptides exhibit bacteriostatic activity towards axenically grown *M. avium*

**Figure 4:**
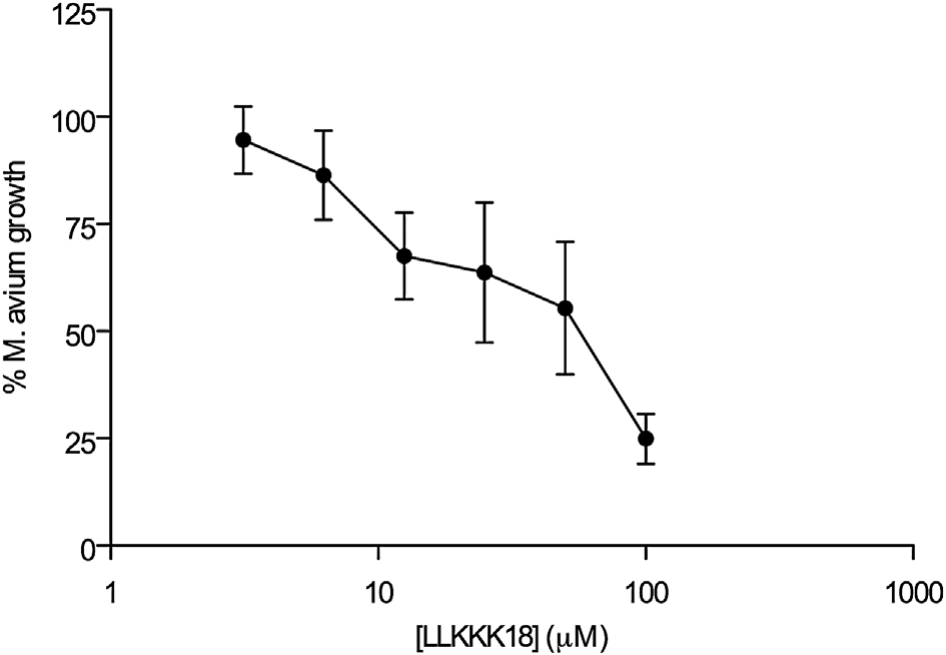
Dose–response curve of the inhibition of *M. avium* growth vs. peptide concentration. The IC_50_ values were calculated from the represented curve, as the concentration needed to inhibit by 50% the mycobacterial growth. The mean ± SEM for each concentration is represented considering the results obtained in triplicates for four independent experiments.

To investigate if *M. avium* is susceptible to the antimicrobial peptide cathelicidin, the bacterium was grown in vitro in 7H9 broth in the presence of increasing concentrations of a CRAMP analogue. As shown in Figure 4, the peptide restricted in a dose-related manner the growth of *M. avium* with a concentration of 58.81 ± 0.07 µM leading to a 50% reduction in growth (means ± SEM from four independent experiments).

### Role of CRAMP in the control of *in vivo* infections with *M. avium*

**Figure 5:**
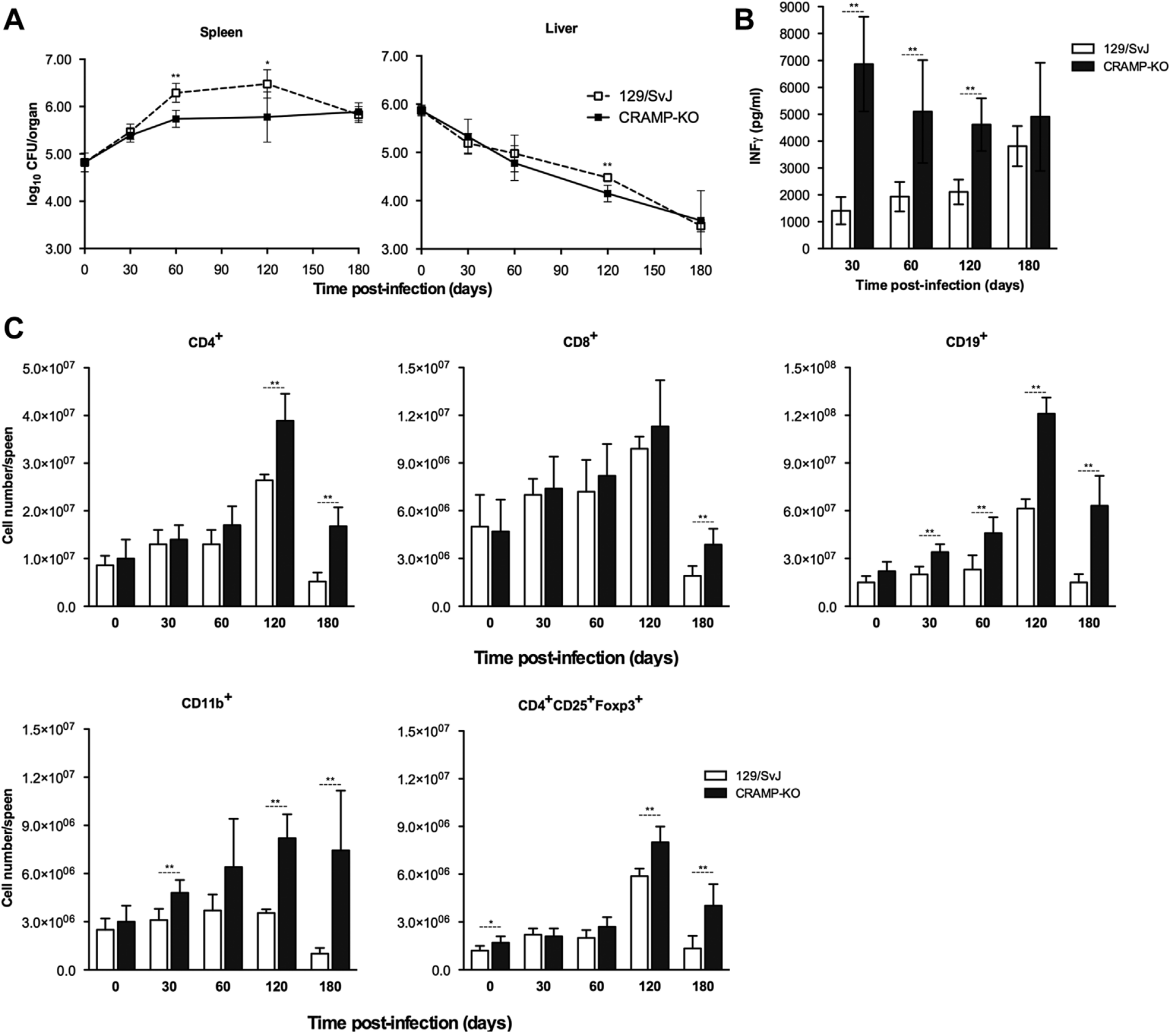
Characterisation of the infection and immune response following an intravenous challenge of 129/SvJ and CRAMP-KO mice with *M. avium* 2,447. Bacterial growth in the spleen and liver of control 129/SvJ and CRAMP-KO animals (A). IFN-γ secreted by spleen cells from 129/SvJ or CRAMP-KO mice infected with *M. avium* 2,447, after in vitro stimulation for 72 h with *M. avium* antigens (B). Supernatants were collected and IFN-γ was quantified by ELISA. Number of splenic cells expressing CD4, CD8, CD19, CD11b or CD4CD25Foxp3 in the different time points of infection (C), as analysed by flow cytometry. Data are represented as the mean value ± SD from three to five animals per group, from one representative experiment out of a total of two experiments.

We next investigated the *in vivo* role of murine cathelicidin in *M. avium* infection. CRAMP-deficient mice were intravenously infected with 10^6^ CFU of *M. avium* (strains 2,447 (intermediate virulence) or 25,291 (high virulence)) and the mycobacterial infection was followed in the liver and spleen for up to 180 days and compared to that in 129/SvJ control animals. *M. avium* 2,447 was shown to be slowly eliminated from the livers of both CRAMP-deficient and wild-type mice at similar rates in both strains (Fig. 5A). In the spleen, after an initial 60-day period of proliferation, the growth of this bacterial strain was arrested. Such control of infection was slightly and transiently improved in the spleen of CRAMP-deficient mice as compared to control animals. Spleen cells from CRAMP-deficient mice responded to mycobacterial antigens with superior IFNγ production as compared to cells from control animals (Fig. 5B). CRAMP-deficient mice had a greater expansion of splenic CD4^+^ T cells at late time-points of infection when compared to control animals (Fig. 5C) and also showed increased numbers of B cells, myeloid CD11b^+^ cells and regulatory (CD4^+^CD25^+^FoxP3^+^) T cells.

**Figure 6:**
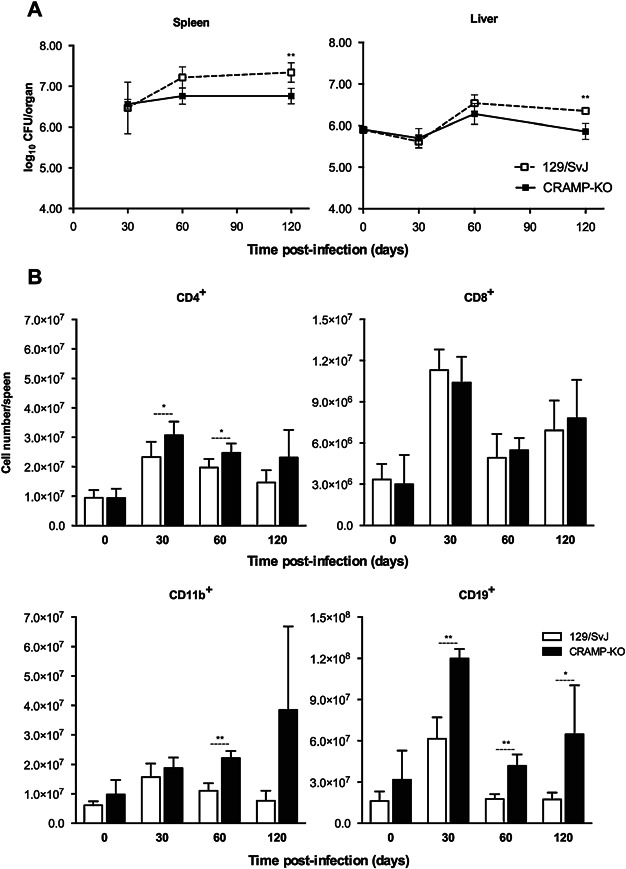
Characterisation of the infection and immune response following an intravenous challenge of 129/SvJ and CRAMP-KO mice with *M. avium* 25,291. Bacterial growth in the spleen and liver of control 129/SvJ and CRAMP-KO animals (A). Number of splenic cells expressing CD4, CD8, CD19 or CD11b in the different time points of infection (B), as analysed by flow cytometry. Data are represented as the mean value ± SD from three to five animals per group of one experiment.

Similar data were obtained for the highly virulent strain 25,291 showing an improved splenic control of the infection (Fig. 6A) and enhanced expansion of lymphocytes (Fig. 6B) in CRAMP-deficient mice as compared to control animals.

## Discussion

We started by analysing the expression of the cathelicidin-coding gene in murine macrophages infected with *M. avium*. Infection of BMMφ, TLR2/6 activation or treatment with TNF, all led to an increase of *Camp* mRNA levels. In order to get insights into the mechanism by which *M. avium* induces cathelicidin expression, we showed that TLR2 or TNF knockdown strongly impaired *M. avium*-induced *Camp* expression. Additionally, in FSL-1 stimulated TNF-null BMMφ, cathelicidin expression is highly reduced when compared to wild-type cells. Given that *M. avium* is a TLR2 agonist [28] and that *Camp* mRNA expression upon bacterial infection depends on TLR2 activation, our data suggest that TNF production after TLR2 stimulation by *M. avium* is crucial to induce murine cathelicidin expression.

TLR2 stimulation leads to the recruitment of a series of adaptor molecules that lead to the activation of NF-κB [29]. Previous findings showed that the promoter of the mouse cathelicidin-coding gene contains predicted binding sites for this transcription factor [11], and more recently NF-κB was shown to induce CRAMP expression in murine mast cells [30]. Although we did not address the role of NF-κB on *M. avium*-induced transcription of *Camp*, it is probable that this transcription factor mediates the effect of TLR2 activation on *Camp* expression. It should be noted that there is no NF-κB response element in the human cathelicidin-encoding gene [31], but rather multiple vitamin D receptor elements (VDREs) that are not found in the murine counterpart [32]. This is consistent with the observed absence of CRAMP induction by the active form of vitamin D_3_ in mouse cells [33] and also with the fact that mice, as nocturnal animals, evolved in such a way as to not respond to vitamin D_3_ synthesis upon solar exposure. These observations point to clear differences in the regulation of the human and mouse cathelicidin-encoding genes. However, TLR2 triggering in human cells may still lead to activation of cathelicidin expression via TNF induction, something which is worth looking at. In our experiments, TNF is only partly responsible for the transcription of the gene possibly because TLR2 signalling is acting directly as well. TNF can be seen as an autocrine or paracrine enhancer of such expression. This may differ in human cells. We also observed residual induction of cathelicidin expression in the absence of TLR2 suggesting that other receptors in macrophages, possibly signalling via NF-κB, may have a minor role in promoting the expression of this gene.

We also show here that the cathelicidin-related antimicrobial peptide is not required for the control of *M. avium* growth in mouse macrophages despite the observation that its expression is effectively induced upon contact with mycobacteria or bacteriostasis-inducing cytokines or TLR2-agonists. These data might be consistent with an endogenous resistance of this mycobacterial species to this antimicrobial molecule. However, we found that an analogue of LL-37, the human version of this peptide, is active against *M. avium* 2,447 (Fig. 4). This raises the possibility that endogenous CRAMP may not reach the compartment inhabited by the mycobacterium. Future work will address whether the intracellular targeting of the mycobacteria by the peptide is the basis of the present observations or whether infection modulates peptide production at post-transcriptional levels. These in vitro data were corroborated by the *in vivo* infection experiments that showed that CRAMP-deficient mice were not more susceptible to infection. Unexpectedly, however, a slight improvement of bacterial control was observed in the spleen of CRAMP-deficient mice as compared to wild-type controls. This was paralleled by an exacerbation of the inflammatory responses in the former animals as compared to the latter. Thus, we detected an increased IFN-γ response, an augmented CD4^+^ T cell expansion and an increased accumulation of different populations of leukocytes in the spleen of CRAMP-deficient animals as compared to their controls. We conclude that not only does CRAMP fail to act as an effector antimicrobial mechanism against *M. avium* in mouse macrophages as it limits inflammation mediated by the immune response.

Cathelicidin-derived peptides have already been shown to have immunomodulatory activities (reviewed in [34]). Cathelicidin peptides may act on phagocytic cells either promoting [35] or reducing [36] their pro-inflammatory activity, promoting Th1 responses [37]. *Camp*-deficient mice had altered *in vivo* B and T cell responses to model antigens [38]. Using an *in vivo* mouse model of infection, we showed here that the inhibitory effects of CRAMP dominate over potentially adjuvant effects. We postulate that if we can directionally target these peptides to phagosomes, cathelicidin-derived peptides may serve not only to help eliminate the pathogen but to limit pathology associated with mycobacterial disease. This will require proper encapsulation in a delivery system that will reach phagosomes within macrophages residing in granulomas.

Although we centred our in vitro studies on macrophages, other cell types produce cathelicidin. Most prominently, neutrophils are the richer source for these proteins/peptides, which are stored within their specific granules [39]. Though, neutrophils are not the major phagocytes recruited to the mycobacterial granuloma although they may be found there and could donate their content to the infected macrophage [40]. However, depletion of neutrophils or neutrophil granule defects in mice cause an early exacerbation of *M. avium* growth leading to a significant increase in bacterial loads in the first days of infection which is maintained in the ensuing weeks [41], something which was not observed in the CRAMP-deficient mice. It is thus unclear what the cellular source for cathelicidin is in our model. An experimental model of aerogenic tuberculosis infection identified macrophages, bronchial epithelial cells and pneumocytes as the cellular sources or sites of accumulation of most of the cathelicidin induced by infection [42]. It is thus likely that many cells contribute, although it still remains to be known which fraction or fractions will play a role in infection.

## Materials and methods

### Mice and bacterial strains

129/SvJ mice were purchased from Charles River Laboratories, Inc. (Barcelona, Spain), and C57BL/6 mice were bred in our facilities. CRAMP-deficient (CRAMP-KO) mice, on a 129/SvJ background, were bred at the IBMC facilities from a breeding pair provided by Dr. R. Gallo (University of California, USA) [17]. TLR2-deficient mice, on a C57BL/6 background, were bred in our facilities from a breeding pair kindly provided by Drs. O. Takeuchi and S. Akira from the Research Institute for Microbial Diseases (Osaka University, Japan). TNF-deficient mice, on a C57BL/6 background, were purchased from B&K Universal and bred in our facilities. All mice were kept at the IBMC animal facilities, in high efficiency particulate air (HEPA)-filter-bearing cages under 12 h light cycles, and were given sterile chow and autoclaved water ad libitum. All animal experiments were performed in accordance with national and European guidelines for the care and handling of laboratory animals and have been approved by the National Veterinary Board.

The highly virulent *M. avium* strain 25,291, forming smooth transparent (SmT) colonies, was obtained from the American Type Culture Collection (Manassas, VA, USA). Strain 2,447 SmT (isolated from an AIDS patient) was provided by Dr. F. Portaels (Institute of Tropical Medicine, Antwerp, Belgium). *M*. avium strain 2,447 SmT was used unless indicated otherwise in the text. Bacteria inocula were prepared as previously described [28].

### Infection of mice and study of immune cells

Female 129/SvJ and CRAMP-deficient mice aged 8–14 weeks were infected intravenously with 10^6^ CFUs of *M. avium* strains 2,447 or 25,291 through a lateral tail vein. At different time points, animals (*n* = 3–5) were anesthetised with isoflurane and retro-orbital bleeding was performed before sacrifice, also with isoflurane. The livers and spleens were removed in aseptic conditions, homogenised, and serially diluted in distilled sterile water containing 0.05% Tween 80. The resulting suspensions were plated into Middlebrook 7H10 agar medium (BD-Difco, Sparks, MD, USA) supplemented with 10% OADC (oleic acid-albumin-dextrose-catalase) and the number of CFUs counted after 7–9 days of incubation at 37 °C.

Single-cell suspensions from spleens were prepared by teasing portions of the spleen with two sterile slides in DMEM supplemented with 10% FBS. Cells were pelleted by centrifugation, washed with HBSS (Gibco Invitrogen, Paisley, UK), and erythrocytes were lysed with an NH_4_Cl hemolytic buffer. Cell suspension was then washed with HBSS, resuspended in DMEM with 10% FBS, and used for population analysis by flow cytometry or for in vitro stimulation studies. Splenic cells were cultivated at a density of 2 × 10^5^ cells/well in a U-bottom, 96-well cell culture plate and incubated in triplicate in DMEM with 10% FBS, 2 mM l-glutamine (Gibco Invitrogen), 10 mM HEPES (Gibco Invitrogen), 1 mM sodium pyruvate (Gibco Invitrogen), 100 U/mL penicillin 100 µg/mL streptomycin (Gibco Invitrogen), with no further stimulus or in the presence of mycobacterial envelope proteins or Con A (Sigma), both at a concentration of 4 µg/mL. Supernatants from the cultures were collected after 72 h of incubation at 37 °C in a 7% CO_2_ atmosphere, and the IFN-γ produced was quantified by a two-site sandwich ELISA method, using anti-mouse IFN-γ specific antibodies (R4-6A2, BD Pharmingen, San Jose, California, USA, as capture and biotinylated AN-18 as detecting antibody). A standard curve was generated with known amounts of recombinant murine IFN-γ (Genzyme). Mycobacterial envelope proteins were prepared from *M. avium* broth cultures as previously described [43].

### Flow cytometry

For the immunofluorescence staining, 10^6^ splenic cells were incubated in a 96-well plate, for 30 min at 4 °C and in the dark, with FITC-conjugated anti-mouse CD4 (diluted 1:400) and PE-conjugated anti-mouse CD8 (diluted 1:400) antibodies, or with FITC-conjugated anti-mouse CD19 (diluted 1:400) and PE-conjugated anti-mouse CD11b (diluted 1:400) antibodies, in PBS containing 1% FBS. All antibodies were from Biolegend (San Diego, CA, USA). The cells were washed twice with PBS containing 1% FBS, and the analysis of the cell population was based on the acquisition of 20,000 events in a FACSCalibur flow cytometer (BD Biosciences) equipped with CellQuest and FlowJo softwares.

For the analysis of Foxp3 intracellular expression, cells were first stained for the surface molecules, with FITC-conjugated anti-mouse CD4 (diluted 1:400) and PE-conjugated anti-mouse CD25 (diluted 1:400) antibodies (Biolegend), and after fixation and permeablisation, were incubated with an Alexa Fluor 647 conjugated anti-mouse Foxp3 (eBiosciences) based on the manufacturer's recommendations.

### Cell culture

BMMφ were obtained by culturing the bone marrow cells that were flushed from the femurs of male mice with cold HBSS. The resulting cell suspension was centrifuged and the cells gently resuspended in DMEM supplemented with 10 mM l-glutamine, 10 mM HEPES, 1 mM sodium pyruvate, 10% FBS, and 10% L929 cell-conditioned medium (LCCM) as source of M-CSF. To remove fibroblasts, the cells were cultured overnight, at 37 °C in a 7% CO_2_ atmosphere, on cell culture dishes. The non-adherent cells were collected with HBSS, distributed in 24-well plates (5 × 10^5^ cells/well) and incubated at 37 °C in a 7% CO_2_ atmosphere. After 3 days, 10% LCCM was added. On day 7, the medium was renewed with warm DMEM containing 10% FBS and 10% LCCM. On day 10, the cells were fully differentiated into macrophages. For the quantitative analysis of *Camp* mRNA expression in BMMφ and preparation of whole cell extracts, cells were distributed in 6-well plates (2 × 10^6^ cells/well).

### Infection and treatments of BMMφ

On the 10th day of culture, about 10^6^ *M. avium* (∼2 bacteria per macrophage) was added to each well containing the macrophages, in 0.2 mL DMEM. After 4 h of incubation at 37 °C in a 7% CO_2_ atmosphere, cells were washed four times with warm HBSS to remove non-internalised mycobacteria and re-incubated in DMEM with 10% FBS and 10% LCCM. To quantify the number of intracellular mycobacteria, macrophages from triplicate wells were immediately lysed (time zero of infection) with 0.1% saponin (Sigma), and the number of viable bacteria counted as described below. The other cells were incubated for 7 days to measure the intracellular growth of the bacteria. Some macrophages were treated daily with recombinant murine TNF (50 U/mL/day, Peprotech EC, London, UK), during the first 4 days of infection, or treated with fibroblast-stimulating lipopeptide-1 (FSL-1, 0.001 µg/mL, EMC Microcollections, Tübingen, Germany) just after infection. The measurement of mycobacterial growth was done by counting CFUs. Briefly, the cells were lysed by adding 0.1% saponin to each well, the resulting bacterial suspension was 10-fold serially diluted in water containing 0.05% Tween 80, and the dilutions were plated into Middlebrook 7H10 agar plates. The number of colonies was counted after 7–9 days of incubation at 37 °C. For each condition tested, three culture wells were used.

### Isolation of RNA from BMMφ and quantitative RT-PCR analysis for *Camp*

For the quantitative analysis of *Camp* mRNA expression in BMMφ, triplicates were harvested after infection with *M. avium* (mycobacteria added in 0.8 mL DMEM) or after treatment with a single dose of either TNF (50 U/mL) or FSL-1 (0.001 µg/mL). Total RNA was extracted from macrophages using the PureLink™ Micro-to-Midi Total RNA Purification System (Gibco Invitrogen), according to the manufacturer's instructions. Quality of RNA was assessed by determining the OD_260/280_ ratio and by visualisation following agarose gel electrophoresis and ethidium bromide stain. Up to 1 µg of total RNA was transcribed into cDNA, using the RevertAid™ H Minus First Strand cDNA Synthesis kit (Fermentas) according to the recommendation of the manufacturer, with an oligo(dT)_18_ primer. To quantify the gene expression, cDNA for hypoxanthine phosphoribosyltransferase (HPRT) (housekeeping) and CRAMP was amplified using the following primers: HPRT: 5′-GTAATGATCAGTCAACGGGGGAC-3′ (forward) and 5′-CCAGCAAGCTTGCAACCTTAACCA-3′ (reverse); CRAMP: 5′-AAGGAA-CAGGGGGTGGTG-3′ (forward) and 5′-CCGGGAAATTTTCTTGAACC-3′ (reverse). The primers were shown not to co-amplify genomic DNA. A standard curve was generated for each primer pair by using five fourfold dilutions of cDNA from BMMφ, to ensure that PCR efficiency was near 100% and within 5% of each other. All reactions were performed in a total reaction volume of 20 µL, with iQ™ SYBR® Green Supermix (Bio-Rad Laboratories), and carried out in a iQ™ 5 instrument (Bio-Rad Laboratories) with the following parameters: HPRT - 95 °C for 30 s, followed by 40 cycles (91 °C for 10 s, 59 °C for 25 s and 72 °C for 25 s); CRAMP – 92 °C for 30 s, followed by 40 cycles (91 °C for 10 s and 65 °C for 25 s). For each primer pair, a negative control (water) was included during cDNA quantification. After PCR amplification, a melting curve was generated for every PCR product to check the specificity of the PCR reaction. Baseline thresholds were calculated by the Bio-Rad iQ5 program and the threshold cycles (*C*_T_) were used in the Livak (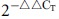) method [44], where *C*_T_ values for CRAMP were normalized to expression levels of HPRT. Data are reported as n-fold changes relative to the control samples.

### Preparation of whole cell extracts and immunoblot analysis

For the isolation of protein from BMMφ, cells were washed twice with ice-cold PBS and lysed with ice-cold RIPA buffer (50 mM Tris–HCl pH 8.0, 150 mM sodium chloride, 1% Igepal, 0.5% sodium deoxycholate, 0.1% SDS, 1 mM EDTA) containing 1:100 protease inhibitors (Sigma) at 4 °C. Adherent cells were scrapped and the cell extracts were centrifuged to remove cell debris. Total proteins were precipitated with trichloroacetic acid and resuspended in electrophoresis sample buffer (50 mM Tris–HCl pH 8.8, 2% SDS, 0.017% bromophenol blue, 10% glycerol, 2 mM EDTA, 100 mM DTT). Equal amounts of protein (30 µg), as defined using the Micro BCA Protein Assay (Pierce) were loaded onto a 12% SDS–PAGE gel, subjected to electrophoresis and transferred to a 0.45 µm nitrocellulose membrane (Amersham Biosciences, Piscataway, NJ, USA) by application of 100 V for 60 min at 4 °C. The membrane was blocked in 5% fat-free milk in PBS-0.1% Tween (PBST) for 1 h at room temperature, and then incubated overnight with a polyclonal rabbit anti-CRAMP(1-39) antibody (Innovagen) diluted 1:500 in PBST with 5% fat-free milk, at 4 °C. Incubation with the secondary antibody, a horseradish peroxidase-conjugated sheep anti-rabbit IgG (The Binding Site, Birmingham, UK) diluted 1:10,000 in PBST with 5% fat-free milk, was performed for 45 min at room temperature. Immunoreactivity was visualized using an enhanced chemi-luminescence (ECL) reagent (Pierce) according to the manufacturer's instructions.

### Determination of LLKKK18 effect on the growth of *M. avium*

Frozen aliquots of *M. avium* strain 2,447 were thawed and diluted to a final concentration of 10^6^ CFU/mL in Middlebrook 7H9 broth medium supplemented with 10% ADC and 0.05% Tween 80. After 2 days (lag phase), the density of a 1:2 dilution of the axenic culture was measured daily at 600 nm until it reached an OD of about 0.132, corresponding to a 0.5 McFarland standard. At this point, the axenic culture was diluted 1:10 in 7H9 broth medium to attain a concentration of 10^7^ CFU/mL and the assay was initiated by adding *M. avium* to a 96-well plate.

The mycobactericidal effect of LLKKK18 (KEFKRIVKRIKKFLRKLV), an analogue of the cathelicidin LL37, was determined by adding serial dilutions (between 3.13 and 100 μM) of this peptide to the axenic culture. The microplate was then incubated in a humidified chamber at 37 °C and the OD measured daily at 600 nm, for up to 7 days. The inhibition of *M. avium* growth at day 7 was expressed as a percentage, towards a control of *M. avium* grown in the absence of LLKKK18, and the concentration needed to reduce mycobacterial growth by 50% (IC_50_) was determined. All measurements were performed in triplicate. Viable mycobacteria at day 7 were also quantified by plating serial dilutions of the suspensions on solid Middlebrook 7H10 agar medium supplemented with 10% OADC. Colony-forming units (CFUs) in each plate were counted 7 days after plating.

### Statistical analysis

Statistically significant differences between groups were determined using the Student's *t*-test (two-tailed, equal variances). Significance was referred like * for *P* < 0.05 and like ** for *P* < 0.01.

## SUPPORTING INFORMATION

**Figure S1.** Expression of the CRAMP-coding gene in BMMφ from C57BL/6 mice, upon TNF stimulation. After a 4 h treatment with 50 U/mL TNF, cells were washed and the levels of *Camp* mRNA were determined by qPCR at the different time points indicated in the graph. Values represent the mean fold expression relative to non-infected or untreated control cells, obtained from triplicate cultures from one representative experiment.
