# Supplementary figures and images for: Endogenous cathelicidin production limits inflammation and protective immunity to Mycobacterium avium in mice
Source: Immun Inflamm Dis. 2013 Oct 31;2(1):1–12. doi: 10.1002/iid3.7 (PMC4220664; doi:10.1002/iid3.7)

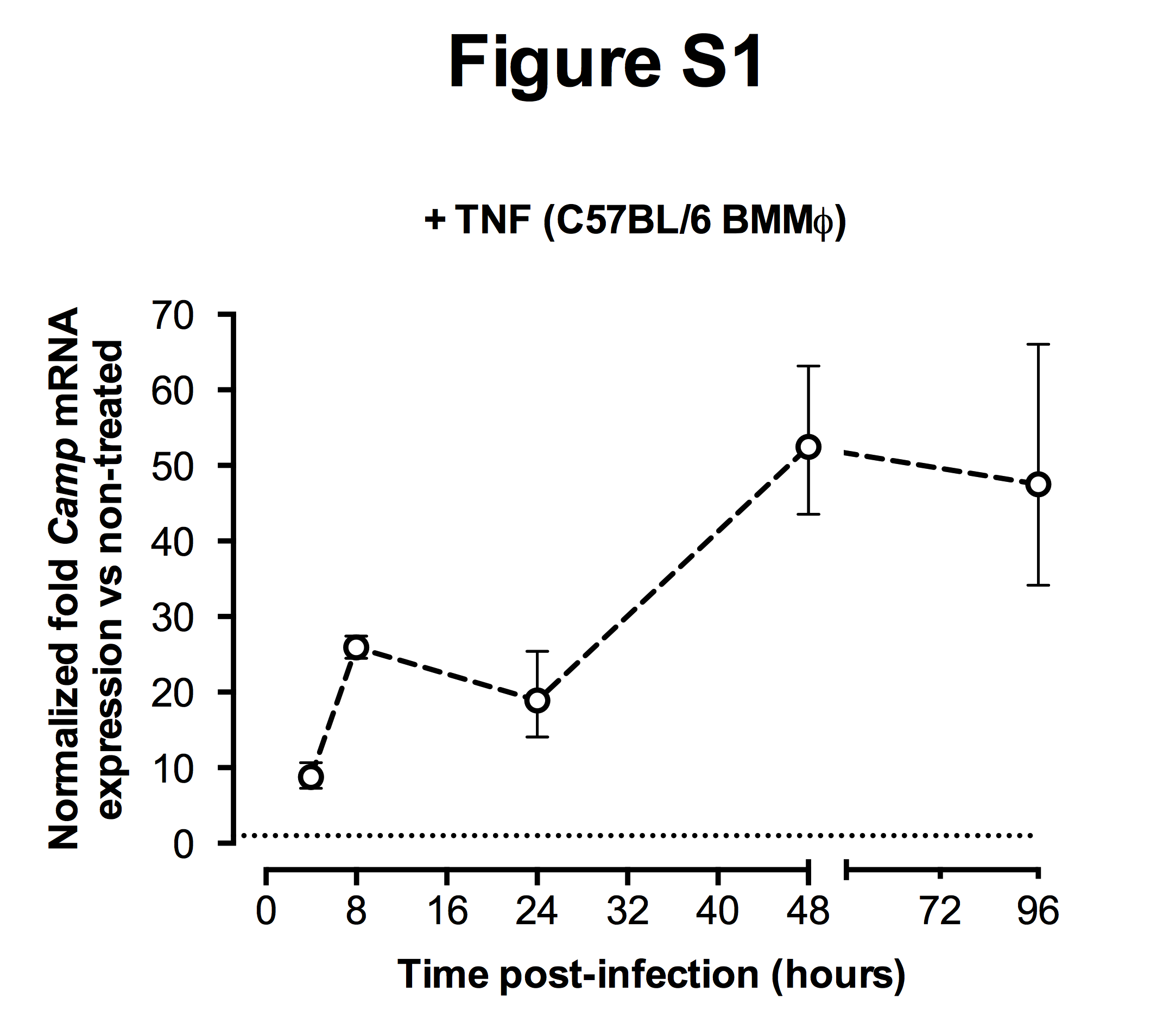

Supplement: Supplementary file 1 — Figure S1. Expression of the CRAMP-coding gene in BMMφ from C57BL/6 mice, upon TNF stimulation. After a 4 h treatment with 50 U/mL TNF, cells were washed and the levels of Camp mRNA were determined by qPCR at the different time points indicated in the graph. Values represent the mean fold expression relative to non-infected or untreated control cells, obtained from triplicate cultures from one representative experiment. [file iid30002-0001-SD1.tif]
